# Correction: Degen et al. Citrinin Exposure in Germany: Urine Biomarker Analysis in Children and Adults. *Toxins* 2023, *15*, 26

**DOI:** 10.3390/toxins15050322

**Published:** 2023-05-06

**Authors:** Gisela H. Degen, Jörg Reinders, Martin Kraft, Wolfgang Völkel, Felicia Gerull, Rafael Burghardt, Silvia Sievering, Jennifer Engelmann, Yvonni Chovolou, Jan G. Hengstler, Hermann Fromme

**Affiliations:** 1 Leibniz Research Centre for Working Environment and Human Factors (IfADo), Ardeystrasse 67, D-44139 Dortmund, Germany; 2 State Agency for Nature, Environment and Consumer Protection North-Rhine Westphalia, Department of Environmental Medicine, Wallneyer Straße 6, D-45133 Essen, Germany; 3 Bavarian Health and Food Safety Authority, Department of Chemical Safety, Toxicology and Exposure Monitoring, Pfarrstraße 3, D-80538 München, Germany; 4 Landeslabor Berlin-Brandenburg, Fachbereich IV-4, Umweltbezogener Gesundheitsschutz, Rudower Chaussee 39, D-12489 Berlin, Germany; 5 Institut und Poliklinik für Arbeits-, Sozial- und Umweltmedizin, Klinikum der Ludwig-Maximilians-Universität München, Ziemssenstraße 1, D-80336 München, Germany

## Error in Table

In the original publication [1], there was a mistake in Table 2. Citrinin exposure assessment based on biomarker results as a range of probable daily intakes (PDI) and expressed as a percentage of the provisional tolerable daily intake (pTDI), as published. The original Table 2 is as follows:

**Table 2:** Citrinin exposure assessment based on biomarker results as range of probable daily intakes (PDI) and expressed as percentage of the provisional tolerable daily intake (pTDI).

Study Group	Probable Daily Intakes (ng per kg Body Weight)			Percentage of the pTDI (i.e., 200 ng/kg bw *)		
	PDI_min_	PDI_median_	PDI_max_	pTDI_min_	pTDI_median_	pTDI_max_
Entire group	2	30	461	1	15	231
Adults (*n* = 138)	2	13	214	1	6.5	107
Children (*n* = 179)	3	214	461	1.5	25	231

* The level of no concern for nephrotoxicity set by EFSA [4].

The value under column of PDI_median_ and the row of Children (*n* = 179) should be corrected. The corrected number should be 50 instead of 214. The corrected Table 2 appears below. The authors state that the scientific conclusions are unaffected. The original publication has also been updated.

**Table 2:** Citrinin exposure assessment based on biomarker results as range of probable daily intakes (PDI) and expressed as percentage of the provisional tolerable daily intake (pTDI).

Study Group	Probable Daily Intakes (ng per kg Body Weight)			Percentage of the pTDI (i.e., 200 ng/kg bw *)		
	PDI_min_	PDI_median_	PDI_max_	pTDI_min_	pTDI_median_	pTDI_max_
Entire group	2	30	461	1	15	231
Adults (*n* = 138)	2	13	214	1	6.5	107
Children (*n* = 179)	3	50	461	1.5	25	231

* The level of no concern for nephrotoxicity set by EFSA [4].
